# Global Trend in Research and Development of CDK4/6 Inhibitors for Clinical Cancer Therapy: A Bibliometric Analysis

**DOI:** 10.7150/jca.51609

**Published:** 2021-04-23

**Authors:** Hongna Lai, Wei Jiang, Jianli Zhao, Xiaoxiao Dinglin, Yudong Li, Shunying Li, Ying Wang, Herui Yao

**Affiliations:** 1 Guangdong Provincial Key Laboratory of Malignant Tumour Epigenetics and Gene Regulation, Sun Yat-Sen Memorial Hospital, SunYat-Sen University, Guangzhou 510120, China.; 2 Breast Tumour Center, Sun Yat-Sen Memorial Hospital, Sun Yat-Sen University, Guangzhou 510120, China.; 3 Department of Pharmacy, Sun Yat-Sen Memorial Hospital, Sun Yat-Sen University, Guangzhou 510120, China.

**Keywords:** CDK4/6 inhibitor, anti-cancer agents, publications, basic research, clinical trial

## Abstract

**Background:** Cyclin-dependent kinase (CDK) 4/6 inhibitors are frequently used anti-cancer agents in hormone receptor-positive breast cancers. This study assessed the course of research and development (R&D) for CDK4/6 inhibitors in terms of publications over the past two decades.

**Methods:** The Web of Science (WOS) and PubMed databases were searched to identify publications related to research on CDK4/6 inhibitors since 2001. The VOS Viewer software was used to analyze co-occurring keywords to stratify the publication data and collaborations in research.

**Results:** There were 1395 publications related to research on CDK4/6 inhibitors since 2001. Eight of the top 10 institutions originated from the USA and the other two were a Swiss Pharmaceutical Company and French Research Institute. Bardia A, the first author for some of the articles published in the USA, was the most prolific with 25 publications. The journal with the most publications was *Cancer Res* with 162 publications. Basic research comprised six of the 10 most frequently cited publications and the rest consisted of three reviews and a clinical trial. The most common keywords for publications since 2011 were “palbociclib”, “abemaciclib”, “ribociclib” and “double blind”, indicating the successful development of CDK4/6 inhibitors as anticancer drugs.

**Conclusions:** This study provides a comprehensive review of the CDK4/6 inhibitors R&D history. The data imply that drug development in this field is a decade-long process and clinical trials have been performed before clinical applications. Thereafter, research was conducted on the adverse effects and drug resistance associated with the inhibitors.

## Introduction

Cyclin-dependent kinases (CDKs) are a family of protein kinases that regulate cell cycle progression 1, 2, gene transcription, mRNA processing, and cell differentiation 3, 4. CDKs are expressed in all known eukaryotes, with evolutionarily conserved regulatory functions to control the cell cycle; for example, yeast can grow normally with the substitution of yeast CDK genes with a homologous human CDK gene 5. In humans, there are at least nine CDKs, four of which (CDK1-4) regulate cell cycle progression 1. CDK4/6 can partner with cyclin D1-3 to direct cell cycle progression. In cells, CDK proteins bind to the corresponding cyclin to form a cyclin-CDK complex and in turn phosphorylate the substrates on serines and threonines, leading to cell cycle progression 1, 2. Thus, the targeting of CDK activities or the formation of the cyclin-CDK complex can effectively inhibit cell cycle progression and cancer cell proliferation. In this regard, during the past 20 years, substantial advancements have been made in research on CDK4/6 inhibitors as anticancer agents 6-9. Among the CDKs, CDK4 and 6 are fundamental drivers of cell cycle progression; after activation by Cyclin D, CDK4/6 phosphorylate and inactivate the retinoblastoma protein (pRb), a cell cycle gatekeeper from the G1 to S phase 10, resulting in a cell cycle transition from the G1 phase to the S phase 11. The maintenance of proliferative signaling through the cell cycle progression is one of the six hallmarks of cancer. “Evading growth suppressors” and “resisting cell death” due to continuous cell cycle progression lead to “replicative immortality”, “angiogenesis”, and “invasion and metastasis”, forming the rest of the six cancer hallmarks proposed by Hanahan and Weinberg in 2000 and 2011 12. In contrast, the development of CDK4/6 inhibitors block Rb inactivation and induce cell cycle arrest at the cell cycle G1 phase 7, 13-17, although a most recent review indicated that CDK4/6 had anticancer effects beyond cell cycle arrest 18. CDK4/5 inhibitor blockage of the CDK4/6 signal pathway may include the inhibition of CDK4/6, Cyclin D, and CDK2 activities 19, 20. Direct blockage of Cyclin D and CDK2 activities can introduce significant side effects and a number of clinical trials using different CDK4/6 inhibitors showed that the inhibition of CDK4/6 activity only was optimal for the control of human cancers 21, 22. Palbociclib developed by Pfizer was approved in 2015 for the treatment of advanced breast cancer that is estrogen receptor-positive (ER+) and human epidermal growth factor receptor-negative (HER2-) in postmenopausal women 23. It was the first small molecule CDK4/6 inhibitor to be approved by the US Food and Drug Administration (FDA). To date, three CDK4/6 inhibitors, i.e., palbociclib, abemaciclib, and ribociclib, have been approved by the US FDA for the treatment of advanced breast cancer 24.

In this study, we assessed publication records from the Web of Science (WOS) and PubMed databases for all research publications on CDK4/6 inhibitors and the course of their development into clinically useful drugs. We utilized Bibliometrics, a series of quantitative tools, to analyze published studies, e.g., the research topics, research status, and publication quality as well as to track the history of research data, the development course of CDK4/6 inhibitors for publication journals, institutions, citations, and the trends in the research. Assessment of the research and development (R&D) history for CDK4/6 inhibitors can benefit small molecule drug R&D by facilitating better understanding of the course, duration, focus, and challenges of research. We expected to provide insightful information about the R&D for these CDK4/6 inhibitors, thereby helping researchers formulate better drug R&D strategies in the future.

## Database Search and Methods

### Search for publications on R&D for CDK4/6 inhibitors

In this study, we first performed a WOS search using the search term “CDK4/6 inhibitor” as the TOPIC and refined the results by selecting the DOCUMENT TYPES “ARTICLE OR PROCEEDINGS PAPER OR REVIEW OR MEETING ABSTRACT” and the LANGUAGE “ENGLISH” (Indexes = SCI-EXPANDED, SSCI, A&HCI, CPCI-S, CPCI-SSH, ESCI, CCR-EXPANDED, IC Timespan = 2001-2020). We next searched PubMed using the search terms “CDK4/6 inhibitor” [MeSH Terms] or “CDK4/6 inhibitor” [All Fields] and (“2001/01/01” [PDAT]: “2019/12/31” [PDAT]) and English [lang]. The literature types included basic research, review, randomized controlled trials, clinical studies, and case reports. To screen for basic research articles, we identified the species as “other animals.” Two of our investigators performed the search and data extraction independently. The titles and abstracts of potentially eligible articles were reviewed and publications unrelated to CDK4/6 inhibitors were excluded. The abstract for each included publication was retrieved, imported into the VOS Viewer software, and analyzed.

### Bibliometric data and analysis

All extracted publications since 2001 were subjected to Bibliometric data construction and analysis. The periodical impact factor (IF) for each journal that published the articles was retrieved from the 2019 periodical citation report database (https://www.medsci.cn/sci/index.do) and citations of each included publication were obtained from WOS tools (http://apps.webofknowledge.com/). For Bibliometric data analyses, we first utilized the functions inherent in the WOS to analyze the research trend and publishing characteristics, including the country/region of origin, publishing institution, total citations, individual average citations, h index, publishing journals, authorship, and research field (basic vs. clinical). We then identified the 10 most cited articles, the top 10 institutions, country of origin, etc. VOS Viewer (Van Eck & Waltman, University of Leiden, Netherlands), Canvus, Microsoft Excel, and other software were used to collate the data and generate graphs for our research findings.

## Results

### Characteristics of CDK4/6 inhibitor-related publications

**Figure 1:**
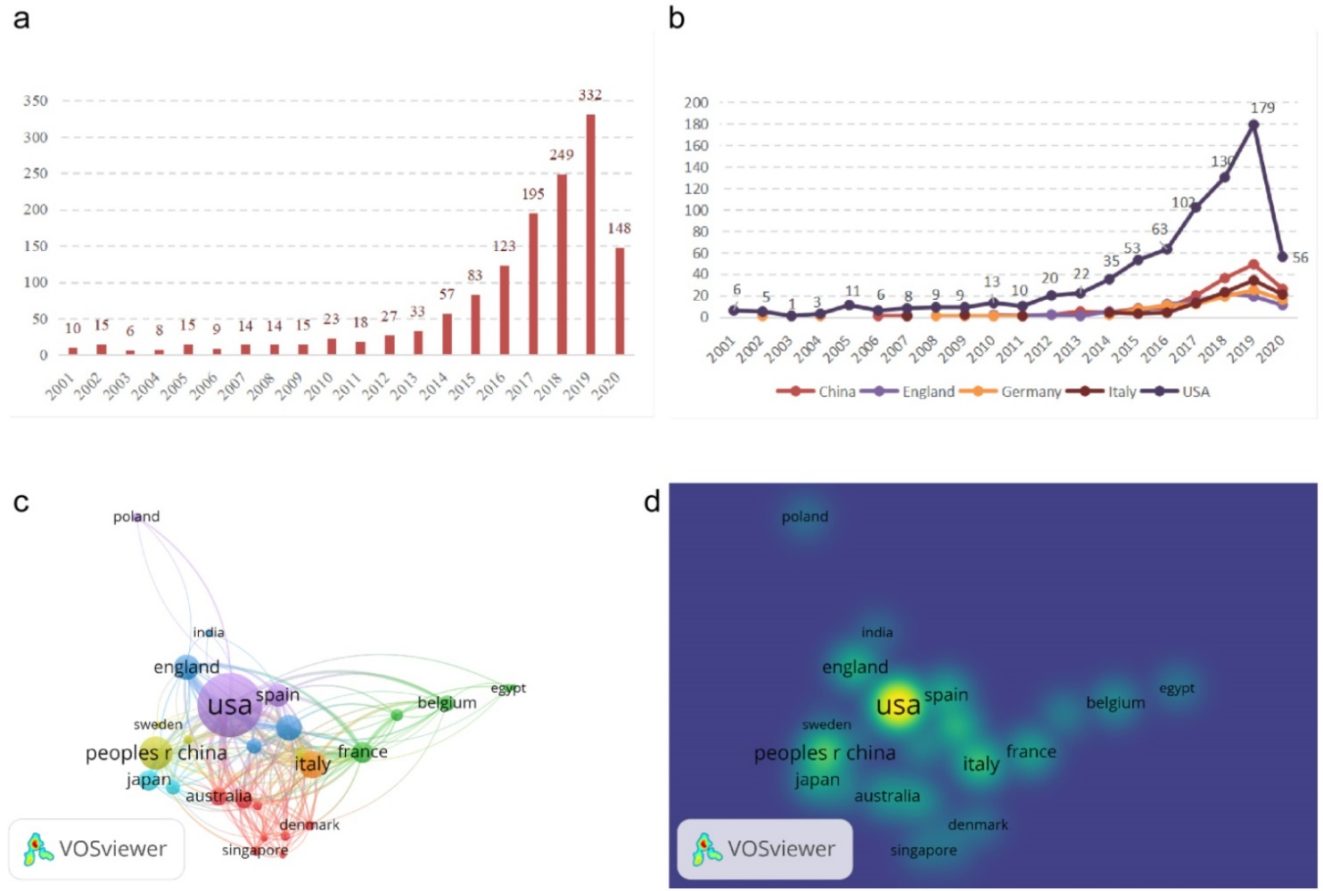
Illustration of CDK4/6 inhibitor-related publications. (a) The annual global number of CDK4/6 inhibitor publications. (b) Time curve of CDK4/6 inhibitor-related publications from the top five countries. (c) Network map of different countries for research on CDK4/6 inhibitors. (d) Density map. Map showing the intensity of different countries in research on CDK4/6 inhibitors.

After searching the WOS and PubMed, we identified 10 publications in 2001 and 332 in 2019, with a total of 1395 qualified articles published since 2001. Thirty-seven countries and regions had studies and publications on CDK4/6 inhibitors, among which the USA ranked the first with 741 articles (53.11%) on R&D for CDK4/6 inhibitors, followed by China (162, 11.61%) and Italy (108, 7.74%). With the exception of the publishing trend in the USA, there was no substantial increase in annual publications related to CDK4/6 inhibitors in other developed countries (Figure 1A & B). There was also a considerable gap in the number of literary works between the USA and other major countries, with publishing peaks in 2019 in all countries.

Furthermore, we performed a network map analysis using VOS Observer and found that the USA was the global center for R&D on CDK4/6 inhibitors and closely coordinated with many other countries, like China, the United Kingdom, and Italy. We also found that Germany had a number of research collaborations with different countries like France, Brazil, and Denmark (Figure 1C & D).

### Most productive organizations and their collaborations

**Figure 2:**
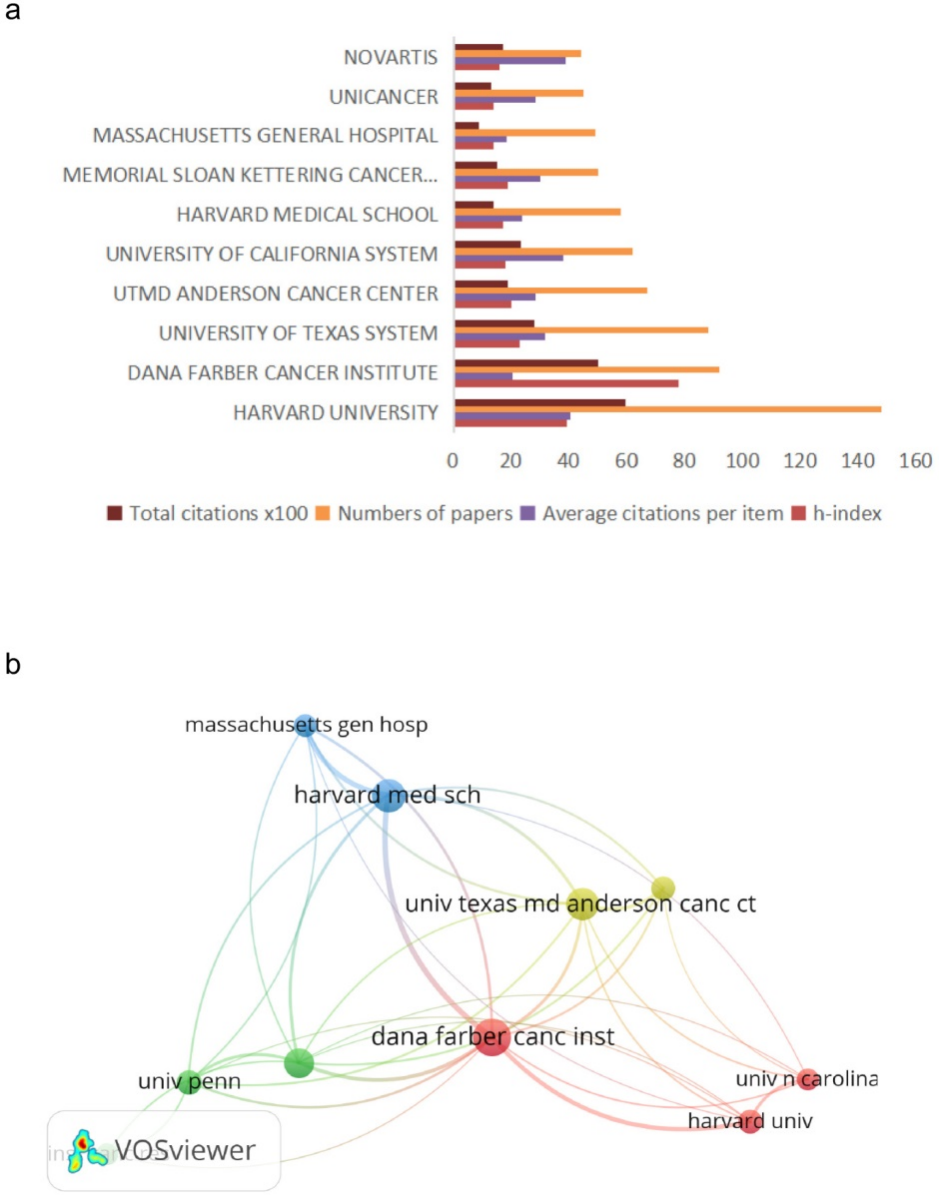
Most productive organizations and their research collaborations on CDK4/6 inhibitors. (a) Most published organizations and their citation reports for research on CDK4/6 inhibitors. (b) VOS Viewer network map. Map analysis showing the collaborations among different organizations for research on CDK4/6 inhibitors.

The 10 most productive organizations are listed in Figure 2. Specifically, Harvard University published 148 articles on R&D for CDK4/6 inhibitors, followed by The Dana Farber Cancer Institute with 92 publications and The University of Texas with 88 publications. Of these top 10 organizations, eight were from the USA and the other two were a Swiss pharmaceutical company and a French research institute. Furthermore, the publications from Harvard University were ranked first with a total of 5974 citations and an average of 40.36 citations per article, for an h index number of 39. There was no significant gap in the article quality between American institutions and other national institutions.

In the VOSviewer analysis, the relationships between University Harvard with its collaboration were visualized. Visualization map of organizations grouped into 4 clusters. It showed that academic cooperative connections among organizations in the USA were relatively concentrated.

### Authorship distributions

**Figure 3:**
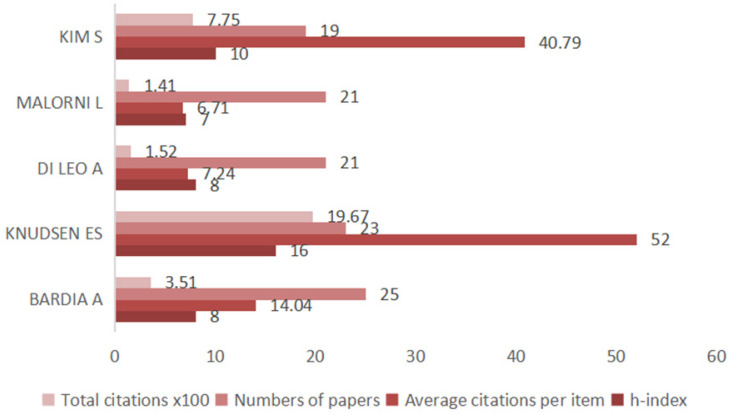
Top five authors in research on CDK4/6 inhibitors. The graph summarizes the five most active first authors and their citations for research on CDK4/6 inhibitors.

A total of 8176 investigators participated in the published research on CDK4/6 inhibitors (Figure 3) and the top three authors were Bardia A at Massachusetts General Hospital, USA, followed by Knudsen ES at Roswell Park Cancer Institute, USA, and DI Leo A at the Hospital of Prato, Italy. Publications from Knudsen ES had the highest number of citations.

### Publishing journals

**Figure 4:**
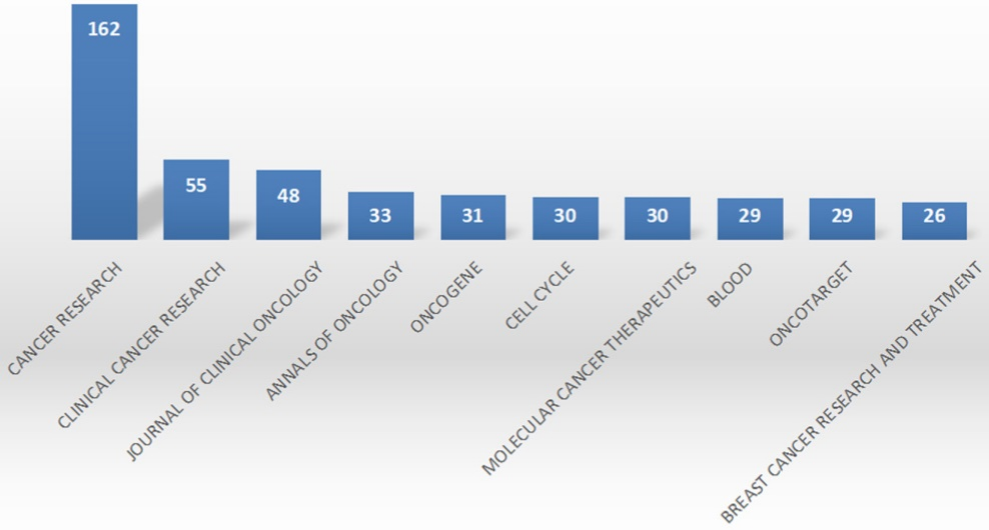
Top 10 journals for the publication of research data on CDK4/6 inhibitors. This graph summarizes the top 10 journals that published research data on CDK4/6 inhibitors.

The top 10 journals published 532 (slightly over 20%) of 1395 articles on CDK4/6 inhibitors R&D (Figure 4). Cancer Res published 162 articles (11.33%), followed by Clin Cancer Res (55, 3.87%) and J Clin Oncol (48, 3.44%). According to the Journal Citation report (2019), Cancer Res, Clin Cancer Res, J Clin Oncol, Ann Oncol, Oncogene, Cell Cycle, Mol Cancer Therap, Blood, and Oncotarget were classified as Q1, while Breast Cancer Research was classified as Q2.

### Research types and categories of CDK4/6 inhibitor R&D

**Figure 5:**
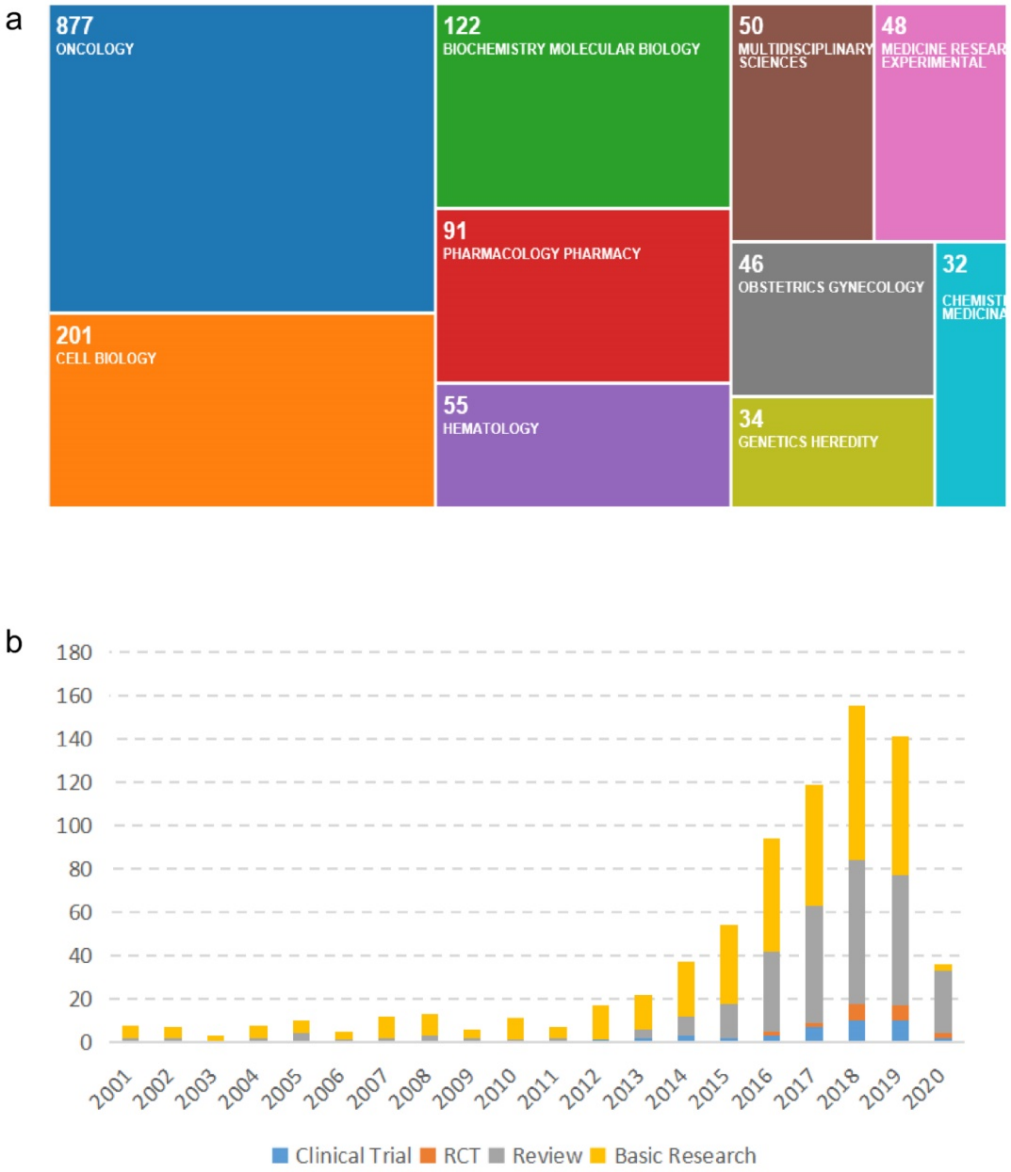
Illustration of the research types and categories for studies on CDK4/6 inhibitors. (a) Categorized worldwide fields for research on CDK4/6 inhibitors. (b) Worldwide distribution of research data on CDK4/6 inhibitors in the past two decades.

The data on the types and categories of publications regarding R&D for CDK4/6 inhibitors are shown in Figure 5. Specifically, there are 65 research categories related to R&D for CDK4/6 inhibitors globally. Oncology is the main research category with a total of 878 (62.93%) publications, followed by Cell Biology (201, 14.4%) and Biochemical Molecular Biology (122, 8.74%). Publications in basic research dominate the R&D for CDK4/6 inhibitors and clinical studies have been increasing annually. Other types of research have maintained a steady growth.

### The top 10 most cited articles on R&D for CDK4/6 inhibitors

We listed the 10 most cited articles in R&D for CDK4/6 inhibitors (Table 1). These top 10 most cited articles were mainly published between 2001 and 2016, with six in basic research, three reviews, and a clinical trial. The most frequently cited article was a basic research article on the cyclin-dependent kinase pathway published by Shapiro G et al in J Clin Oncol. This was followed by “Ribociclib as a first-line treatment for HR-positive advanced breast cancer” published by Hortobagyi GN et al. in New Engl J Med (IF = 74.699).

### Keywords analysis of CDK4/6 inhibitor R&D-related publications

**Figure 6:**
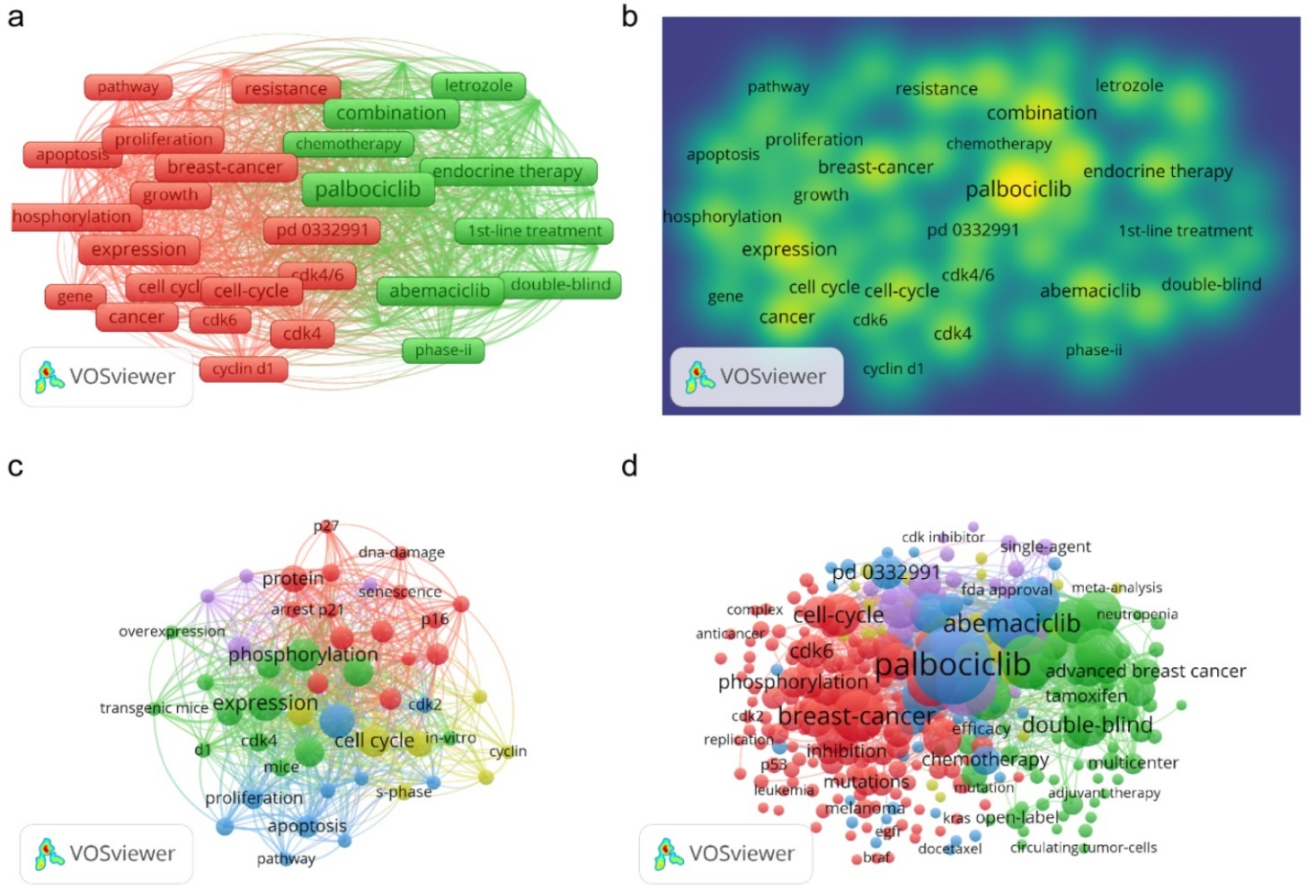
Keyword analytic data for research and publications on CDK4/6 inhibitors. (a) Analysis of co-occurring keywords in the past two decades. (b) Keyword density map for the past 20 years. (c) Analysis of co-occurring keywords in data published between 2001 and 2010. (d) Analysis of co-occurring keywords in data published between 2011 and 2020. The dots represent the keywords and the larger dots indicate a higher occurred frequency of the keywords, while the clusters are labeled using different colors and the links represent the co-occurrence of the keywords.

To search for and identify the trends and directions for R&D on CDK4/6 inhibitors, we used the VOS Viewer software to analyze the distribution of co-occurring keywords (keywords that appeared at least 10 times in the titles and abstracts of all publications). Figure 6 presents 47 such keywords that met the threshold and these keywords were divided into two categories: “treatment” and “anti-tumor activity” (Figure 6A). In the “treatment” group, the most popular keywords were “Palbociclib”, “breast cancer”, and “combination”. In the “anti-tumor activity” group, the most popular keywords were “breast cancer”, “cell cycle”, and “cancer”.

A comparison of 2001 and 2010 showed that the hot topics changed dynamically over time. With the progression of R&D on CDK4/6 inhibitors, more common keywords appeared; for example, in the early stage of CDK4/6 inhibitor R&D (i.e., between 2001 and 2010), there were a few major hot topics, but more keywords on clinical research and treatment, such as “palbociclib”, “abemaciclib”, “ribociclib”, and “double blind” appeared between 2011 and 2020 (Figures 6C & D).

## Discussion

In the current study, we performed a bibliometric analysis (types, citations, journal, institutions, and country of origin) of publications on the scientific research progress in the field of CDK4/6 inhibitors over the past two decades. We found that publications related to CDK4/6 inhibitors rapidly increased by nearly 30 fold since 2001. The USA dominated in CDK4/6 inhibitor R&D, closely followed by China. The study of CDK4/6 inhibitors was mainly focused on oncology, including breast cancer, glioblastoma, and myeloma 25-29. Many basic and clinical studies have confirmed the efficacy of CDK4/6 inhibitors on the treatment of various solid human cancers 25-29. Nevertheless, publications in other areas, such as Nano-medicine research on CDK4/6 inhibitors, are still few and delayed 30, 31. Furthermore, most publications originated from the USA and pharmaceutical companies, such as Pfizer (USA), Novartis (Switzerland), and Eli Lilly (USA) play an important role in R&D for CDK4/6 inhibitors. These companies drive and market CDK4/6 inhibitors in terms of clinical trials and usage in collaboration with different academic institutions and hospitals, which benefit the assessment of the safety and effectiveness of these CDK4/6 inhibitors.

Most journals that published data on CDK4/6 inhibitors were classified as Q1 according to the 2019 journal citation report, indicating that these journals possess a high research and impact value with worldwide popularity. Most of the journals that published data on CDK4/6 inhibitors are in the field of cancer research, especially basic research. Unlike other anti-tumor drugs, there have been many studies of the oncological mechanism and biological signaling pathways for CDK4/6 inhibitors, in line with the development trend of translational medicine 32. With the increase in the number of clinical trials, studies on CDK4/6 inhibitors have also expanded from breast cancer to other types of human cancer 25-29. However, in view of the adverse effects and drug resistance associated with CDK4/6 inhibitors, many drug candidates will be investigated; thus, dominant countries, such as the USA, Europe, and China, should strengthen their support to complete high-quality basic research and clinical trials of CDK4/6 inhibitors.

In the list of the top 10 most cited articles related to R&D on CDK4/6 inhibitors, six were in basic research, three were reviews, and only one was from a clinical trial. At the end of the 20^th^ century, the US National Institutes of Health strengthened and promoted the concept of translational medicine for timely translation of basic research knowledge and findings into the clinical treatment of patients, the improvement of human health, and the eradication of diseases. Translational medicine is committed to bridging the gap between basic research and clinical and public health applications, which indeed provides a revolutionary and novel strategy in drug R&D 33, 34. The characteristics of publications on CDK4/6 inhibitors are consistent with the perception of translational medicine as the right research direction. Our current data also confirmed this trend, with data on CDK4/6 kinase regulation, inhibitors for anti-tumor activity, lab research, and clinical trials. Our findings indicate a change in study keywords over the past 20 years; for example, in the past 20 years, there were 47 threshold recognition keywords related to “treatment” and “anti-tumor activity” between 2001 and 2010, with the emergence of more recent keywords in clinical research and treatment, such as “palbociclib”, “abemaciclib”, “ribociclib”, and “double blind” between 2010 and 2020. Randomized controlled trials are the highest level of evidence-based medicine in the assessment of drug efficacy in patients. In order to verify the antitumor effects of CDK4/6 inhibitors, many randomized controlled trials on CDK4/6 inhibitors have been carried out 26, 28, 35. Therefore, it is not surprising that “double blind” appeared more frequently as a keyword during the last 10 years.

Our current research has some limitations; for example, this analysis only includes English publications; thus, there is a certain degree of selection bias in the study. Moreover, we only searched articles in the WOS and PubMed databases, which could result in the exclusion of other types of publications or databases, leading to incomplete data collection. In addition, for practical reasons, we only imported abstracts from each publication into the VOS Viewer software for the analysis of co-occurring keywords rather than the full-length articles. Further, citations and h-index reports for some articles published in the relevant databases may have been delayed, leading to a system bias in our current study.

## Figures and Tables

**Table 1:** The top ten most cited publications in CDK4/6 inhibitor research

Study title	First authors	Journal	Year	Total citations	Impact factor	Quartile in category
Cyclin-dependent kinase pathways as targets for cancer treatment	Shapiro, GI	J CLIN ONCOL	2006	704	32.956	Q1
Specific inhibition of cyclin-dependent kinase 4/6 by PD 0332991 and associated antitumor activity in human tumor xenografts	Fry, DW, Harvey, PJ	MOL CANCER THERAP	2004	692	5.615	Q1
PD 0332991, a selective cyclin D kinase 4/6 inhibitor, preferentially inhibits proliferation of luminal estrogen receptor-positive human breast cancer cell lines *in vitro*	Finn, RS, Dering, J	BREAST CANCER RES	2009	669	4.988	Q2
The history and future of targeting cyclin-dependent kinases in cancer therapy	Asghar, U	NATURE REVIEWS DRUG DIS	2015	618	64.794	Q1
Ribociclib as First-Line Therapy for HR-Positive, Advanced Breast Cancer	Hortobagyi, G. N.	NEW ENGL J MED	2016	549	74.699	Q1
Cyclin D-dependent kinases, INK4 inhibitors and cancer	Ortega, S	BBA-REV CANCER	2002	537	7.365	Q1
Multiple viral strategies of HTLV-1 for dysregulation of cell growth control	Yoshida, M	ANN REV OF IMMUNOLY	2001	348	19.9	Q1
AKT/PKB phosphorylation of p21(Cip/WAF1) enhances protein stability of p21(Cip/WAF1) and promotes cell survival	Li, Y	J BIOL CHEM		340	4.238	Q2
Cycling to cancer with cyclin D1	Diehl, JA	CANCER BIOL THERAP	2002	328	3.659	Q2
Targeting CDK4 and CDK6: From Discovery to Therapy	Sherr, CJ	CANCER DIS	2016	311	29.497	Q1
